# Supplementary material for: Meta-Analysis and Gene Set Enrichment Relative to ER Status Reveal Elevated Activity of MYC and E2F in the “Basal” Breast Cancer Subgroup
Source: PLoS One. 2009 Mar 9;4(3):e4710. doi: 10.1371/journal.pone.0004710 (PMC2650420; doi:10.1371/journal.pone.0004710)
Supplement: Dataset S1 — GSEA results from first screen. The file contains a zipped archive of the HTML results of the second GSEA screen. Reports for enriched or depleted datasets may be accessed from the “index.html” link within the my_analysis.GseaPreranked.1219975950625.rpt.zip ZIP archive. Enrichment plots are available for each of the 50 most enriched and depleted gene sets. (13.32 MB ZIP) [file pone.0004710.s001.zip › my_analysis.GseaPreranked.1219975950625.rpt.zip!//V$E2F1_Q3.html]

Details for gene set V$E2F1\_Q3[GSEA]

|  || Dataset | zSco.ERneg.to.ERpos\_collapsed |
| Phenotype | NoPhenotypeAvailable |
| Upregulated in class | na\_pos |
| GeneSet | V$E2F1\_Q3 |
| Enrichment Score (ES) | 0.48101914 |
| Normalized Enrichment Score (NES) | 2.1186185 |
| Nominal p-value | 0.0 |
| FDR q-value | 9.236561E-5 |
| FWER p-Value | 0.0040 |
Table: GSEA Results Summary

  

Fig 1: Enrichment plot: V$E2F1\_Q3      
 Profile of the Running ES Score & Positions of GeneSet Members on the Rank Ordered List

  

| PROBE | GENE SYMBOL | GENE\_TITLE | RANK IN GENE LIST | RANK METRIC SCORE | RUNNING ES | CORE ENRICHMENT || 1 | E2F3 |  |  | 6 | 7.369 | 0.0210 | Yes |
| 2 | NASP |  |  | 59 | 6.201 | 0.0351 | Yes |
| 3 | PIM1 |  |  | 78 | 6.025 | 0.0513 | Yes |
| 4 | AK2 |  |  | 85 | 5.971 | 0.0682 | Yes |
| 5 | KLF5 |  |  | 87 | 5.966 | 0.0855 | Yes |
| 6 | GPRC5B |  |  | 103 | 5.845 | 0.1014 | Yes |
| 7 | MCM6 |  |  | 198 | 5.317 | 0.1098 | Yes |
| 8 | GMNN |  |  | 206 | 5.302 | 0.1247 | Yes |
| 9 | ORC1L |  |  | 212 | 5.280 | 0.1397 | Yes |
| 10 | USP1 |  |  | 222 | 5.212 | 0.1542 | Yes |
| 11 | PAX6 |  |  | 224 | 5.208 | 0.1693 | Yes |
| 12 | TFRC |  |  | 259 | 5.071 | 0.1815 | Yes |
| 13 | CDC25A |  |  | 281 | 4.993 | 0.1944 | Yes |
| 14 | MCM7 |  |  | 289 | 4.970 | 0.2083 | Yes |
| 15 | STMN1 |  |  | 310 | 4.907 | 0.2211 | Yes |
| 16 | EZH2 |  |  | 312 | 4.888 | 0.2353 | Yes |
| 17 | ARHGAP11A |  |  | 361 | 4.763 | 0.2455 | Yes |
| 18 | SNRPD1 |  |  | 440 | 4.552 | 0.2528 | Yes |
| 19 | DMD |  |  | 481 | 4.406 | 0.2626 | Yes |
| 20 | TYRO3 |  |  | 516 | 4.316 | 0.2726 | Yes |
| 21 | PLK4 |  |  | 524 | 4.292 | 0.2846 | Yes |
| 22 | MCM2 |  |  | 630 | 4.080 | 0.2885 | Yes |
| 23 | MCM3 |  |  | 656 | 4.019 | 0.2983 | Yes |
| 24 | NCL |  |  | 678 | 3.977 | 0.3083 | Yes |
| 25 | ABCF2 |  |  | 764 | 3.816 | 0.3130 | Yes |
| 26 | TCP1 |  |  | 779 | 3.799 | 0.3230 | Yes |
| 27 | TMPO |  |  | 792 | 3.788 | 0.3331 | Yes |
| 28 | ILF3 |  |  | 801 | 3.774 | 0.3435 | Yes |
| 29 | MSH2 |  |  | 819 | 3.748 | 0.3531 | Yes |
| 30 | ABCE1 |  |  | 882 | 3.649 | 0.3590 | Yes |
| 31 | NUTF2 |  |  | 1044 | 3.419 | 0.3567 | Yes |
| 32 | NOL4 |  |  | 1077 | 3.383 | 0.3642 | Yes |
| 33 | NOLC1 |  |  | 1222 | 3.217 | 0.3626 | Yes |
| 34 | DNAJC9 |  |  | 1243 | 3.198 | 0.3704 | Yes |
| 35 | CDC2 |  |  | 1259 | 3.182 | 0.3785 | Yes |
| 36 | POLD1 |  |  | 1260 | 3.182 | 0.3878 | Yes |
| 37 | FBXO5 |  |  | 1304 | 3.137 | 0.3936 | Yes |
| 38 | PCNA |  |  | 1346 | 3.087 | 0.3995 | Yes |
| 39 | SLITRK5 |  |  | 1356 | 3.070 | 0.4078 | Yes |
| 40 | ING3 |  |  | 1398 | 3.042 | 0.4135 | Yes |
| 41 | EPHB2 |  |  | 1441 | 3.007 | 0.4191 | Yes |
| 42 | BAT3 |  |  | 1509 | 2.933 | 0.4225 | Yes |
| 43 | ATAD2 |  |  | 1532 | 2.917 | 0.4294 | Yes |
| 44 | EPHB1 |  |  | 1675 | 2.780 | 0.4267 | Yes |
| 45 | NUP62 |  |  | 1684 | 2.770 | 0.4341 | Yes |
| 46 | E2F1 |  |  | 1695 | 2.764 | 0.4414 | Yes |
| 47 | HIRA |  |  | 1696 | 2.763 | 0.4495 | Yes |
| 48 | PKMYT1 |  |  | 1715 | 2.753 | 0.4561 | Yes |
| 49 | SUMO1 |  |  | 1736 | 2.736 | 0.4626 | Yes |
| 50 | UGCGL1 |  |  | 1779 | 2.709 | 0.4673 | Yes |
| 51 | CASP8AP2 |  |  | 1930 | 2.587 | 0.4634 | Yes |
| 52 | RRM2 |  |  | 2047 | 2.485 | 0.4619 | Yes |
| 53 | H2AFZ |  |  | 2105 | 2.447 | 0.4647 | Yes |
| 54 | SFRS7 |  |  | 2226 | 2.370 | 0.4624 | Yes |
| 55 | POLR2A |  |  | 2274 | 2.334 | 0.4657 | Yes |
| 56 | SOAT1 |  |  | 2362 | 2.281 | 0.4657 | Yes |
| 57 | IPO7 |  |  | 2413 | 2.247 | 0.4684 | Yes |
| 58 | MRPL18 |  |  | 2469 | 2.217 | 0.4707 | Yes |
| 59 | KCNA1 |  |  | 2559 | 2.169 | 0.4703 | Yes |
| 60 | SYT11 |  |  | 2584 | 2.156 | 0.4747 | Yes |
| 61 | HNRPD |  |  | 2585 | 2.156 | 0.4810 | Yes |
| 62 | DNMT1 |  |  | 2844 | 2.017 | 0.4673 | No |
| 63 | ATF5 |  |  | 2941 | 1.962 | 0.4657 | No |
| 64 | PELP1 |  |  | 2968 | 1.951 | 0.4694 | No |
| 65 | RASAL2 |  |  | 3067 | 1.901 | 0.4675 | No |
| 66 | SSBP3 |  |  | 3087 | 1.891 | 0.4716 | No |
| 67 | CDC6 |  |  | 3185 | 1.847 | 0.4696 | No |
| 68 | C10ORF119 |  |  | 3202 | 1.838 | 0.4737 | No |
| 69 | C9ORF86 |  |  | 3333 | 1.779 | 0.4690 | No |
| 70 | PAQR4 |  |  | 3434 | 1.736 | 0.4665 | No |
| 71 | SMAD6 |  |  | 3436 | 1.736 | 0.4715 | No |
| 72 | TOPBP1 |  |  | 3475 | 1.722 | 0.4736 | No |
| 73 | SLCO3A1 |  |  | 3563 | 1.679 | 0.4719 | No |
| 74 | PIAS1 |  |  | 3798 | 1.587 | 0.4587 | No |
| 75 | PRPS1 |  |  | 3889 | 1.556 | 0.4564 | No |
| 76 | POLE2 |  |  | 3975 | 1.520 | 0.4544 | No |
| 77 | AP1S2 |  |  | 4154 | 1.452 | 0.4451 | No |
| 78 | FOXO3A |  |  | 4159 | 1.448 | 0.4490 | No |
| 79 | STAG2 |  |  | 4224 | 1.421 | 0.4483 | No |
| 80 | RQCD1 |  |  | 4253 | 1.412 | 0.4503 | No |
| 81 | HNRPUL1 |  |  | 4310 | 1.387 | 0.4501 | No |
| 82 | GRIA4 |  |  | 4341 | 1.377 | 0.4518 | No |
| 83 | CLSPN |  |  | 4483 | 1.324 | 0.4450 | No |
| 84 | GABRB3 |  |  | 4549 | 1.303 | 0.4438 | No |
| 85 | CACNA1G |  |  | 4739 | 1.239 | 0.4331 | No |
| 86 | KIAA0101 |  |  | 4770 | 1.230 | 0.4344 | No |
| 87 | CORT |  |  | 4877 | 1.196 | 0.4298 | No |
| 88 | SMG1 |  |  | 4882 | 1.194 | 0.4330 | No |
| 89 | HNRPR |  |  | 4913 | 1.185 | 0.4341 | No |
| 90 | ATP1B4 |  |  | 4975 | 1.164 | 0.4329 | No |
| 91 | ARHGAP6 |  |  | 4991 | 1.159 | 0.4351 | No |
| 92 | MYH10 |  |  | 5076 | 1.124 | 0.4320 | No |
| 93 | FLI1 |  |  | 5103 | 1.114 | 0.4333 | No |
| 94 | EED |  |  | 5267 | 1.060 | 0.4240 | No |
| 95 | ANAPC10 |  |  | 5626 | 0.937 | 0.3995 | No |
| 96 | CCNT1 |  |  | 5647 | 0.931 | 0.4007 | No |
| 97 | GCH1 |  |  | 5810 | 0.875 | 0.3910 | No |
| 98 | ADAMTS2 |  |  | 5988 | 0.817 | 0.3799 | No |
| 99 | YBX2 |  |  | 6299 | 0.716 | 0.3585 | No |
| 100 | ALDH6A1 |  |  | 6317 | 0.711 | 0.3592 | No |
| 101 | GRIK2 |  |  | 6357 | 0.699 | 0.3583 | No |
| 102 | SHMT1 |  |  | 6406 | 0.681 | 0.3567 | No |
| 103 | SPTB |  |  | 6641 | 0.609 | 0.3406 | No |
| 104 | POLD3 |  |  | 6667 | 0.601 | 0.3405 | No |
| 105 | TFAP4 |  |  | 6777 | 0.561 | 0.3339 | No |
| 106 | ADCY8 |  |  | 6840 | 0.539 | 0.3307 | No |
| 107 | ASCL1 |  |  | 6905 | 0.517 | 0.3274 | No |
| 108 | ZIM2 |  |  | 7151 | 0.436 | 0.3100 | No |
| 109 | RBBP4 |  |  | 7160 | 0.432 | 0.3107 | No |
| 110 | TBX6 |  |  | 7723 | 0.223 | 0.2686 | No |
| 111 | SNPH |  |  | 7912 | 0.142 | 0.2548 | No |
| 112 | SP3 |  |  | 7914 | 0.141 | 0.2551 | No |
| 113 | APRIN |  |  | 7954 | 0.121 | 0.2525 | No |
| 114 | PEG3 |  |  | 8018 | 0.099 | 0.2480 | No |
| 115 | MAML3 |  |  | 8034 | 0.091 | 0.2471 | No |
| 116 | MEIS1 |  |  | 8167 | 0.051 | 0.2372 | No |
| 117 | SIX5 |  |  | 8286 | 0.006 | 0.2283 | No |
| 118 | SLC9A5 |  |  | 8303 | 0.000 | 0.2271 | No |
| 119 | MXD3 |  |  | 8312 | -0.002 | 0.2265 | No |
| 120 | ANKHD1 |  |  | 8331 | -0.007 | 0.2251 | No |
| 121 | HIST1H2BK |  |  | 8356 | -0.020 | 0.2234 | No |
| 122 | SYNGR4 |  |  | 8638 | -0.147 | 0.2024 | No |
| 123 | FMO4 |  |  | 8775 | -0.199 | 0.1927 | No |
| 124 | SFRS1 |  |  | 8891 | -0.255 | 0.1847 | No |
| 125 | DCK |  |  | 8935 | -0.280 | 0.1822 | No |
| 126 | ID3 |  |  | 8993 | -0.308 | 0.1788 | No |
| 127 | PPM1D |  |  | 9078 | -0.350 | 0.1735 | No |
| 128 | SLC25A3 |  |  | 9309 | -0.459 | 0.1573 | No |
| 129 | CNOT3 |  |  | 9334 | -0.476 | 0.1569 | No |
| 130 | FLJ25476 |  |  | 9347 | -0.481 | 0.1574 | No |
| 131 | PCSK1 |  |  | 9351 | -0.483 | 0.1586 | No |
| 132 | MAZ |  |  | 9415 | -0.521 | 0.1553 | No |
| 133 | ZCCHC8 |  |  | 9511 | -0.566 | 0.1497 | No |
| 134 | ASXL2 |  |  | 9736 | -0.689 | 0.1347 | No |
| 135 | GSPT1 |  |  | 9841 | -0.751 | 0.1290 | No |
| 136 | SALL1 |  |  | 9959 | -0.818 | 0.1225 | No |
| 137 | HIST1H1D |  |  | 10475 | -1.139 | 0.0867 | No |
| 138 | XTP3TPA |  |  | 10592 | -1.236 | 0.0815 | No |
| 139 | C11ORF30 |  |  | 10656 | -1.284 | 0.0804 | No |
| 140 | HMGN2 |  |  | 10916 | -1.492 | 0.0651 | No |
| 141 | C14ORF130 |  |  | 10945 | -1.523 | 0.0674 | No |
| 142 | AP4M1 |  |  | 11123 | -1.683 | 0.0589 | No |
| 143 | PHF15 |  |  | 11215 | -1.755 | 0.0571 | No |
| 144 | HOXC10 |  |  | 11345 | -1.878 | 0.0527 | No |
| 145 | FHOD1 |  |  | 11408 | -1.941 | 0.0537 | No |
| 146 | GPS2 |  |  | 11528 | -2.058 | 0.0506 | No |
| 147 | LASP1 |  |  | 11661 | -2.179 | 0.0470 | No |
| 148 | HIST1H2BI |  |  | 11761 | -2.279 | 0.0461 | No |
| 149 | USP52 |  |  | 11931 | -2.478 | 0.0405 | No |
| 150 | EIF4B |  |  | 11934 | -2.480 | 0.0475 | No |
| 151 | TMEM15 |  |  | 12137 | -2.754 | 0.0402 | No |
| 152 | CXORF12 |  |  | 12144 | -2.762 | 0.0478 | No |
| 153 | RPS6KA5 |  |  | 12285 | -2.949 | 0.0458 | No |
| 154 | NELL2 |  |  | 12593 | -3.420 | 0.0324 | No |
| 155 | TBX3 |  |  | 12795 | -3.880 | 0.0284 | No |
| 156 | ARID4A |  |  | 12806 | -3.906 | 0.0390 | No |
Table: GSEA details [plain text format]

  

Fig 2: V$E2F1\_Q3: Random ES distribution      
 Gene set null distribution of ES for **V$E2F1\_Q3**
